# Enhanced Harmonics Reactive Power Control Strategy Based on Multilevel Inverter Using ML-FFNN for Dynamic Power Load Management in Microgrid

**DOI:** 10.3390/s22176402

**Published:** 2022-08-25

**Authors:** Harun Jamil, Faiza Qayyum, Naeem Iqbal, Do-Hyeun Kim

**Affiliations:** 1 Department of Electronics Engineering, Jeju National University, Jejusi 63243, Korea; 2 Department of Computer Engineering, Jeju National University, Jejusi 63243, Korea; 3 Advanced Technology Research Institute, Jeju National University, Jejusi 63243, Korea

**Keywords:** renewable energy resource, passive filters, synchronous reference frame control, feed forward neural network, sine pulse width modulation

## Abstract

The shift of the world in the past two decades towards renewable energy (RES), due to the continuously decreasing fossil fuel reserves and their bad impact on the environment, has attracted researchers all around the world to improve the efficiency of RES and eliminate problems that arise at the point of common coupling (PCC). Harmonics and un-balance in 3-phase voltages because of dynamic and nonlinear loads cause a lagging power factor due to inductive load, active power losses, and instability at the point of common coupling. This also happens due to a lack of system inertia in micro-grids. Passive filters are used to eliminate harmonics at both the electrical converter’s input and output sides and improve the system’s power factor. A Synchronous Reference Frame (SRF) control method is used to overcome the problem related to grid synchronization. The sine pulse width modulation (SPWM) technique provides gating signals to the switches of the multilevel inverter. A multi-layer feed forward neural network (ML-FFNN) is employed at the output of a system to minimize mean square error (MSE) by removing the errors between target voltages and reference voltages produced at the output of a trained model. Simulations were performed using MATLAB Simulink to highlight the significance of the proposed research study. The simulation results show that our proposed intelligent control scheme used for the suppression of harmonics compensated for reactive power more effectively than the SRF-based control methods. The simulation-based results confirm that the proposed ML-FFNN-based harmonic and reactive power control technique performs 0.752 better in terms of MAE, 0.52 for the case of MSE, and 0.222 when evaluating based on the RMSE.

## 1. Introduction

One of the most eminent insurgencies of the 21st century is how to manage the ever-rapid increasing stipulation of electrical energy. As natural resources continue to deplete worldwide, power generation will become increasingly expensive [1]. Due to the intermittent and inertia-less nature of renewable energies (REs), concerns arise related to stability, the integration of micro-grids with the main power grid, and how to eliminate problems that occur at the point of common coupling (PCC) [2]. Because of the presence of various power electronic devices, one of them is the voltage source converter (VSC), which is most commonly used in interconnecting AC grids and integrating large-scale REs. Excessive use of non-linear devices causes a substantial increase in the issues related to power quality in power electronic frameworks [3].

Multi-level inverter technology has become a very important alternative due to recent breakthroughs in the field of high-power medium-voltage energy control. An electrical network with medium voltage is generally coupled to governed ac actuators in the megawatt range. It is not easy to directly integrate a single power semiconductor switch with medium voltage grids (2.3, 3.3, 4.16, or 6.9 kV). A novel family of multi-level inverters has been created in order to operate at higher voltage levels [4]. Many medium-voltage applications today use diode-clamped multi-level inverters with an IGBT and forced-air cooling. These applications cover numerous high-power loads, such as conveyors, blowers, compressors, pumps, and fans. Multi-pulse rectifiers have traditionally been used to lower harmonics in line current. Due to the drive’s high power and the dc link’s capacitive filters, which worsen the input current’s distortion, the introduction of current harmonics into the power supply complicates the application of these inverters. To eliminate harmonics, transformers are used for phase shifting in these multi-pulse rectifiers (12-pulse, 18-pulse, etc.). Multi-level rectifiers have been recommended as a replacement for phase shift transformers. The use of the NPC inverter and its expansion to a multi-level converter was identified in [5].

- They are able to produce output voltages with reduced dv/dt and extremely low distortion;
- They have very low distortion when drawing input current;
- They produce less common-mode voltage (CM), which lessens the strain on the motor bearings. Additionally, CM voltages can be removed utilizing advanced modulation techniques [6];
- They are capable of running at lower switching frequencies.

The device voltage level is effectively doubled by the NPC inverter without the necessity for precise voltage matching. To illustrate the benefits of NPC inverters for utility applications and motor drive, two significant patents [7,8] were filed. Since then, the cascade inverter has attracted a lot of interest because of the rising need for medium-voltage high-power inverters. Multilevel inverters are widely employed in medium and high-power voltage applications nowadays. Mills, conveyors, blowers, pumps, Laminators, compressors, fans, and other devices are only a few of the field uses.

Harmonic and reactive power compensation is ideal for diode-clamped and cascaded multilevel inverters [9,10]. Diode-clamped multilevel inverters also contain equivalently distinct dc sources to facilitate power conversion involving real power, such as in motor drives. On the other hand, the diode clamped multilevel inverter can balance its dc voltage without the need for additional isolated power sources, making it the optimum choice for utility applications such as harmonic/reactive compensation [11]. The diode clamped multilevel inverter for reactive power generation, and compensation (STATCOM) has been made available for purchase by GEC Alsthom T&D [12].

Voltage-source inverters (VSIs), which operate at a voltage level between 11 and 16 kV, or commonly 13.8 kV, are in high demand. Currently, source converters, which employ thyristor devices with a built-in reverse voltage blocking capability, dominate the power electronics industry for distribution and transmission voltage levels. Thyristors’ slow switching speeds and inability to gate off are their principal drawbacks. The extensive use of VSIs in distribution voltage levels can be easily anticipated due to gate-turn-off high-voltage semiconductor devices in multilevel inverters.

The power conversion circuits used as an energy module or building block in distributed energy systems, often those utilizing alternative energies such as solar panels and fuel cells, can be simply designed with a distinct source connected through them to produce individual output. Multiple modules can then be configured on a diode clamped inverter. A cost-effective method can be used to build the system, and it can provide isolation without the use of a transformer.

A non-linear parameter-varying system with load disturbance can be controlled intelligently using an artificial neural network (ANN). The design of such a system may not necessitate the use of a mathematical model [13]. Although proportional integral (PI) and proportional integral derivative (PID) controllers are frequently employed for motor control applications, when control settings, loading circumstances, and the motor itself is modified, they do not produce adequate results. Fuzzy logic (FL) has recently been very popular in various control applications. Without knowing the specific model of the system, the FL controller can be designed. Even if the motor’s characteristics change, stable functioning is ensured by the fuzzy logic controller (FLC) design methodology [14]. The primary drawback of a fuzzy control system is the substantial computational work required to convert language control principles into matching control actions. For industrial applications, ANN-based intelligent control systems have been rapidly expanding [15]. It has been discovered that ANN-based intelligent control strategies are more effective and easier to use in control applications [16]. The superiority of neural controllers over fuzzy controllers for microprocessor implementation has been demonstrated in [17]. Due to their rapid computation rate, ANNs are increasingly being used in DC drive applications [18]. This paper compares the converter behavior for both PI and ANN controllers in great detail. The system’s small signal modelling is the foundation for the PI controller’s design. The system’s small signal modelling serves as the foundation for the PI controller’s design. A range of operating circumstances, such as a step change and a modulation variation used as a change in load, are tested to see how well the controller’s works. The steady-state error and the response’s rising time were chosen as the parameters for comparison. It is demonstrated that ANN-based controllers outperform PI speed controllers in terms of rising time and steady-state error. It also significantly minimizes the total harmonic as compared to other conventional techniques such as active–passive filters. This benefit results from the generalization ability of the neural network and the smooth control surface of the neural controller.

The power quality issues encountered by low and medium power systems are often addressed using shunt active power filter (SAPF)-based solutions that have a simple structure [19]. However, the performance of the conventional PI controller within the stationary frame exhibits poor performance that leads to infinite gain for DC signals. In this regard, the scientific community has presented several solutions for APF, including proportional resonant (PR) control, hysteresis control, etc. [3]. Among the existing control strategies [20], Synchronous Reference Frame (SRF) theory is used to produce the compensating current. Furthermore, a detailed schematic flow of a multilevel converter is presented for a micro-grid connected inverter under dynamic load. The block diagram includes a 3-phase main electrical grid, dynamic load, SRF control, control for operating switches of a multilevel converter, and an artificial intelligence system for the compensation of harmonics and controlling reactive power. In addition, the proportional integral controller assists in handling the loops and steady-state errors [21].

This research study focuses on the applications pertaining to power electronic converter controllers and machine learning techniques to ensure the quality of the electrical power system in terms of harmonics, synchronization, and reactive power losses. So, a systematic modelling method, which is missing in the literature, should consider these important things.

The main contributions of the proposed study are delineated below:

- Five-level diode clamped voltage source converter (MLDC-VSC) is developed to connect renewable energy resources to the main electrical grid;
- Synchronous reference frame control (SRFC) technique is employed to cater to unbalanced three-phase voltages, mitigate harmonics, and synchronize the voltages and frequencies of the micro-grid with the main electrical grid, which vary due to dynamic load;
- Passive filters are employed as low-pass filters to remove higher-order harmonics from the electrical system;
- Finally, to improve the approximation of non-linearities produced by inverter switches and dynamic load, a multi-layer feed-forward neural network (ML-FFNN) is used to reduce MSE by adaptively varying the ML-FFNN weights using a stochastic gradient descent algorithm;
- In addition, various simulation results and analyses are presented as a proof-of-concept to highlight the significance of the proposed ML-FENN-based compensation of reactive power.

Furthermore, the SIMULINK toolbox simulates the control methods in MATLAB. The simulation outcomes revealed that less distortion in current waveforms is achieved using ANN. The rest of the structure is organized as follows: Section 2 introduces existing approaches related to reactive power compensation and its comparison with our proposed approach. Section 3 introduces the control algorithms used in system configuration. Section 4 shows the implementation of the ANN and optimization algorithm for harmonic and reactive power compensation. Section 5 discusses the simulation results and analysis. Limitations of our proposed study is presented in Section 6. Section 7 concludes the whole paper with possible future directions.

## 2. Literature Review

Commercial-grade semiconductor technology development and optimization have considerably impacted the creation of a brand-new generation of superior and reliable power converters. Even when put next to assail JFET devices [22], SiC–BJT-based power converters are reported to perform well. The basic disadvantage is that these devices have poor gain and should be activated with an oversized base current [23]. As a result, the bottom drive circuit becomes more advanced, lowering potency and moving converter performance considerably [24]. Multi-terminal HVDC systems and DC grids are presently in demand for installing multi-terminal DC-DC converters [25]. On the other hand, most recent DC-DC converters work at low power levels. Moreover, many power unit (MW) converters share inherent weaknesses, such as an absence of fault isolation capabilities, in keeping with recent reports. Ref. [26] offers a topology for a change of magnitude DC-DC converter, achieving terribly high DC voltage stepping ratios and MW-level power transfer. Furthermore, the converter was updated in [27] to allow power reversal and to function in step-down mode.

Power converters, drives, and renewable energy are now widely used in the energy trade due to advancements in power physical science [28]. Several typologies are given to meet the assorted desires of commercial applications. There are four general power conversion varieties: AC-AC, AC-DC, DC-DC, and DC-AC [29,30,31]. In Ref. [32], a novel bidirectional, isolated, single-stage AC-DC converter is planned for dq current management. Previous work has achieved open unity loop power issue exploitation with the innovative design. However, it lacks reactive power regulation and depends on a non-distorted voltage wave shape to realize unity power issues. Making calculations significantly simpler is the primary goal of transforming the three-phase instantaneous voltages and currents into the synchronously rotating reference dqo frame. The second benefit is that it gives the system operator separate control over the current’s reactive (q-axis) and active (d-axis) components. The modulation system is increased with a phase shift part, permitting the phase and magnitude of the converter’s input current to be controlled. Randomized modulation techniques are well-liked thanks to their ability to cut back on switching-frequency harmonics in single-phase converters [33]. These schemes are used to remove the harmonics and unfold them into the frequency spectrum by randomizing one or additional modulation parameters, such as the change frequency, the output pulse position, or the output pulse dimension, leading to low peak spectral power of the input current harmonics and output voltage harmonics. However, randomized modulation techniques have the disadvantage of obtaining a larger output ripple noise voltage when put next to PWM. Ref. [34] describes a brand-new delta-sigma modulated greenhouse emission converter that employs a pulse area modulation approach. The authors improve the delta-sigma modulation approach with the quality PWM system to reduce the current rating of change devices. The authors additionally conducted a performance comparison of the proposed and previous modulation techniques. In Ref. [35], among the modulation ways under discussion are randomized pulse dimension modulation, randomized pulse position modulation, and randomized carrier modulation with mounted and variable duty cycle.

In another article, the prime focus of the study was on satisfactoriness and theme relevancy in AC-DC converters; the impact of randomization on the effectiveness of spreading the dominating frequencies in constant frequency pulse dimension modulation schemes. The proposed management strategy is compared to a conventional PI controller, and the PWM modulation technique for the control of converter switches [36]. A single-carrier modulation technique was proposed in [37] for standard structure converters as an improved carrier part placement modulation strategy for modular multilevel converter topology (MMC). It employs one triangular carrier to make multi-layer modulation. Its smart harmonic characteristics are for carrier part disposition modulation and switch balanced carrier part shifted modulation distribution. To tackle the matter of MMC sub-module electrical condenser voltage imbalance, this work proposes a change allocation technique supported by sub-module electrical condenser voltage sorting. Finally, we look at the value and run current of a transformer-less star electrical converter with a three-level neutral-point-clamping topology [38].

## 3. Proposed Methodology

**Figure 1:**
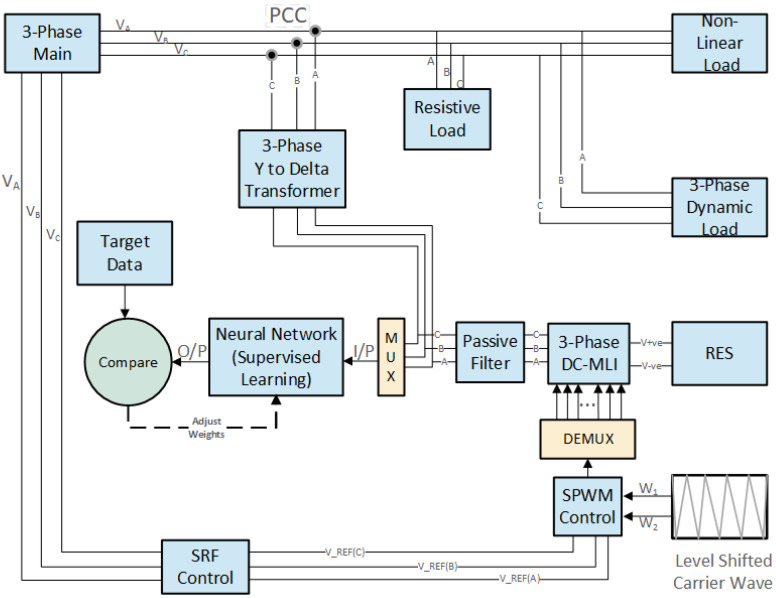
Proposed scheme for artificial intelligence-based grid tied inverter.

In Figure 1, starting from the block diagram Renewable Energy Source (RES), dc electrical energy is converted into AC electrical energy with the help of Diode Clamped Multilevel Inverter.

Passive filters are used here to mitigate the transients produced by Inverter switches and also compensate for current and voltage ripples. From the output of passive filters, 3-phase Y to Delta step-up transformer is used to connect the micro-grid with the main electrical grid. The point where two electrical systems are connected is called (PCC) point of common coupling. Resistive, nonlinear and 3-phase dynamic load is connected with the main electrical grid. At the point of common coupling, the problem of voltage unbalance occurs. The Synchronous Reference Frame (SRF) control technique is used to cater for this issue. In this 3-phase reference, voltages are taken from the main grid and fed into the input of SRF. From here, 3-phase stationary reference frame is converted into two orthogonal reference frames, Alpha-Beta, with the help of Clark transformation. Further, these two Alpha-Beta components are placed orthogonally on the rotor reference frame through Park-transformation, and the representation is called dq-representation. DQ representation greatly simplifies the control of AC machines. There are controllers such as PI, which are designed to track DC references rather than AC sinusoidal references. From here, these dq-component steady-state errors are removed and converted into Alpha-Beta components with the help of inverse Park transformation and from Alpha-Beta to a 3-phase stationary reference frame with the help of inverse Clark transformation. That is how unbalanced voltages at the point of PCC are removed.

Now the output of DCMLI is used to train the neural network using supervised learning. In this, the target data are known, and the neural network’s output is compared. Using the batch gradient descent algorithm, the neural network weights are changed to eliminate the error. This optimization algorithm is used to increase the network performance.

### 3.1. Power Conversion

Power supply is available commercially and domestically through transmission and distribution service providers (TDSPs) or electric distribution utilities (EDUs). Electronic systems require power, and the power supply maintains the electrical system. Selecting the appropriate power supply can be the crucial difference between a device working at an optimal level, and it may also deliver uncertain results. Different loads require distinct power supplies, i.e., single-phase and three-phase AC loads require regulation; variable DC for speed control required for DC motors; integrated circuits; microprocessors require voltages ranging from 15 V, 5 V, 3.3 V, 1.5 V, etc.; and X-ray and scanning machines draw power pulses. We need power conversion because there is an imparity between the power supply available and the power supply required by the loads. The conventional power conversion schemes do not work for the above examples. This requires rectification and inversion mechanisms.

### 3.2. Three Phase Diode Clamped Multilevel Voltage Source Inverter

Three-phase multi-level converter typologies, such as diode-clamped inverter topology (DCIT), achieved considerable consideration in renewable energy systems. For high DC and AC voltage in grid connected mode or residential applications multilevel DCIT are widely utilized as an interface for RES.

**Figure 2:**
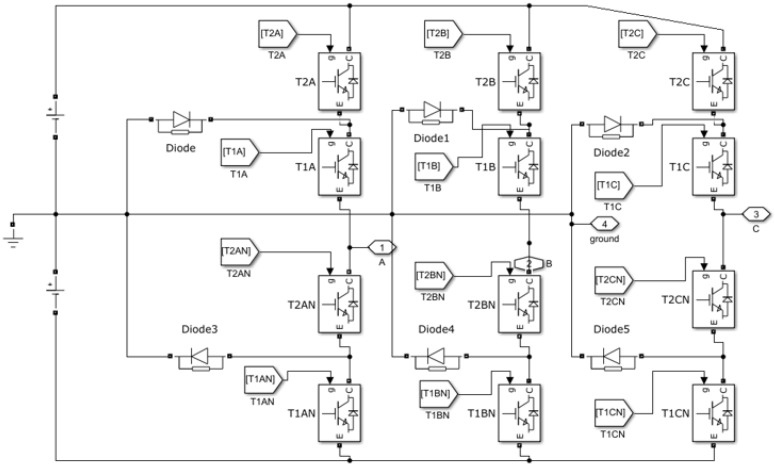
Three phase diode clamped multilevel voltage source inverter.

**Figure 3:**
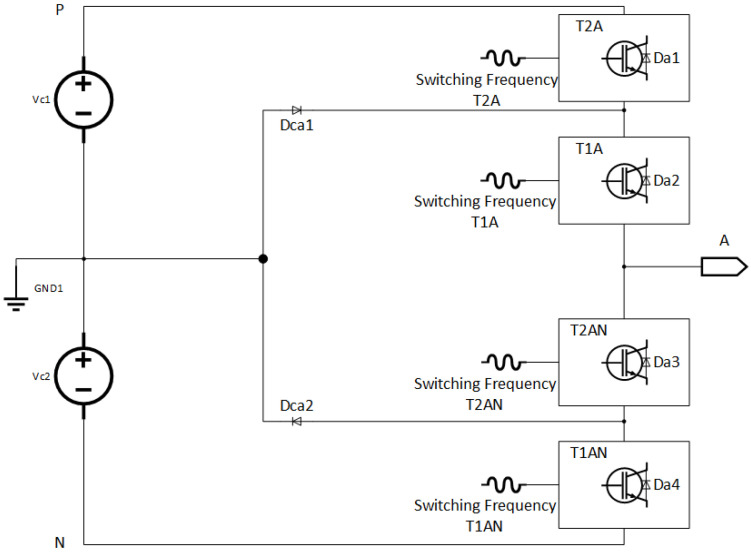
Single leg of three phase diode clamped multilevel inverter.

First, the basic operation of DCIT is shown in Figure 2, which is analyzed with equal DC link voltages and gives a brief overview of the switching states and the practical operations of the converter. Figure 3 depicts the leg structure of a positive half of a five-level DCIT, where the voltages across the DC capacitors are $V_{c1}=V_{c2}=\frac{V_{dc}}{2}$ and the total DC link voltage is $V_{dc}$. By using pair of DC capacitors, $C_{1}$ and $C_{2}$, the DC bus voltage is divided into two voltage sources. Through diodes $D_{6}$ and $D_{3}$ equal DC link voltages maintained across the Capacitors. The switching of switches in DCIT is so that the top switches (T1A, T2A) work in a complementary manner to the bottom switches (T2AN, T1AN). The single and three-phase formations can be formed by paralleling two and three converter branches. For additional output voltage, a converter can be constructed by adding a pair of switching devices, extra DC link sources, diodes and capacitors.

Anti-parallel diodes are used in IGBTs to work in different load power factors. By using the gating signals, bidirectional flow for positive and negative load current is made possible. The switching states of a single switch are represented as “1” and “0”, which are “high ” and “low”, respectively. Voltage levels can be synthesized at the output, If the neutral point *N* of DC link voltage is regulated at half of the total input DC voltage, which is $\frac{V_{dc}}{2}$.

The switching state of both top switches T2A=0,T1A=0 in time duration $\left(0<t\leq t_{1}\right)$ are “off” so that their complements (T2AN and T1AN) are “on”. When the load current is positive, reverse diodes ($D_{a3}$ and $D_{a4}$) conduct. However, in the negative load, current (T2AN and T1AN) conduct and the current loop consist of (T2AN and T1AN) through the load.

Similarly, the positive and negative load current paths are when the switching state T2A=0, T1A=1 in time duration $(t_{1}$ < *t* $\leq t_{2})$. Hence, the complementary switches in this leg (T2AN and T1AN) are “on” and “off”, respectively. When the IGBT T1A and the anti-parallel diode $D_{ca1}$ conduct, it means positive voltage polarity across the load, and the current direction is positive. Therefore, the current loop, which consists of $C_{2}$, $D_{ca1}$ and T1A, can discharge $C_{2}$. On the other hand, for the negative load current, the conduction of anti-parallel diode $D_{ca2}$ and IGBT T2AN depends upon the direction of current and voltage polarity. The current loop consists of $C_{2}$, $D_{ca2}$, and T2AN in the negative load current, also it charges $C_{2}$ through the load. The output voltage in this switching state ($t_{1}$ < *t* $\leq t_{2})$ is: $V_{an}\left(t\right)=V_{c1}=\frac{V_{dc}}{2}$.

Likewise, switching state T2A=1, T1A=1 in time ($t_{2}$ < *t* ≤ $t_{3}$) are “on”, so that the complementary switches T2AN and T1AN, are “off”. When the load current is positive, the switches T2A and T1A conduct and the current loop consists of $C_{1}$, $C_{2}$, T2A and T1A. However, $D_{a1}$ and $D_{a2}$ conduct for the negative load current and the current flows through $C_{1}$, $C_{2}$, $D_{a1}$, and $D_{a2}$. Therefore, the output voltage in ($t_{2}$ < *t* ≤ $t_{3}$) is: $V_{an}\left(t\right)=V_{c1}+V_{c2}=V_{dc}$.

The parameters in Table 1, such as current regulator, reactive and active power control are used for the implementation of a field oriented controller, which is used for reactive power compensation. The switching frequency denotes the on/off switching speed of a DCMLI. Shunt and series capacitance and inductance values are used for passive filters for the suppression of harmonics arises due to the nonlinear load in the electrical system.

### 3.3. Control Algorithm

To cater for the unbalance in three-phase voltages at PCC, transients are produced due to the IGBTs in a converter and provide adequate biasing of converter switches to minimize the harmonics and increase synchronization; several control algorithms are used to enhance the results.

#### 3.3.1. Switching with SPWM Control Technique

Many methods in the literature use the sinusoidal pulse width modulation (SPWM) control technique because of its popularity in multi-level power conversion typologies. It is simple to implement and also has the advantage of low IGBT losses. The input to the SPWM module is the level-shifted carrier waves.

#### 3.3.2. SRFC Algorithm

Synchronous reference frame control algorithm is good for three-phase control applications. SRFC helps us to eliminate the controller for every three phases. Instead, keep to two controllers (P and Q) for all three phases. The benefit of what is achieved here using SRFC is we deal with dc quantities. For control applications, dc quantities are easily controllable without having steady-state errors.

a. Clark’s Transformation: In this 3-phase reference, voltages are taken from the main grid and fed into the input of SRFC. From here 3-phase stationary reference frame is converted into two orthogonal reference frame Alpha-Beta with the help of Clark-transformation as shown in Equation (1):

(1) $$i_{\alpha \beta \gamma }\left(t\right)=Ti_{abc}\left(t\right)=\sqrt{\frac{2}{3}}\left[\begin{array}{ccc} 1 & -\frac{1}{2} & -\frac{1}{2}\\ 0 & \frac{\sqrt{3}}{2} & -\frac{\sqrt{3}}{2}\\ \frac{1}{\sqrt{2}} & \frac{1}{\sqrt{2}} & \frac{1}{\sqrt{2}} \end{array}\right]\left[\begin{array}{c} i_{a}\left(t\right)\\ i_{b}\left(t\right)\\ i_{c}\left(t\right). \end{array}\right]$$

b. Phase-locked loop (PLL): To predict 3-phase main electrical grid voltages, in the case of grid-tied RES, we need to track phase angle and grid frequency. PLL is used to estimate the phase angle of the main electrical grid. It overcomes the error generated due to the difference in phase angle between the input and output of the phase lock loop. A comparator with a low pass filter is used to estimate the phase. The error at the angle detector output can be fed to the discrete PI controller to remove the steady-state error. This control is performed in dq-representation because controllers such as PI are designed to track dc-references rather than ac sinusoidal signals.

c. Park Transformation: Two Alpha-Beta components are placed orthogonally on the rotor reference frame through Park transformation, and the representation is called dq-representation. DQ representation greatly simplifies the control of AC machines. From here, these dq-components’ steady state error removes. Rotation matrix Equation (2), converting 2-phase orthogonal stationary reference voltages into d−q orthogonal rotatory reference voltages.

(2) $$\left[\begin{array}{c} d\\ q \end{array}\right]=\left[\begin{array}{cc} \cos(wt) & \sin(wt)\\ -\sin(wt) & \cos(wt). \end{array}\right]\times \left[\begin{array}{c} \alpha \\ \beta \end{array}\right]$$

d. Inverse Park Transformation: here, these dq-component’s steady state error removes and converted into Alpha-Beta components with the help of inverse Park transformation. Rotation matrix Equation (3), converting 2-phase orthogonal rotatory reference voltages into alpha-beta orthogonal stationary reference voltages.

(3) $$\left[\begin{array}{c} \alpha \\ \beta \end{array}\right]=\left[\begin{array}{cc} \cos(wt) & -\sin(wt)\\ \sin(wt) & \cos(wt) \end{array}\right]\times \left[\begin{array}{c} d\\ q. \end{array}\right]$$

e. Inverse Clark Transformation: From Alpha-Beta stationary reference voltages to 3-phase stationary reference Frame with the help of inverse Clark transformation. Rotation matrix Equation (4), converting 2-phase orthogonal stationary reference voltages into 3-phase 120 degree electrical apart stationary voltages.

(4) $$i_{abc}\left(t\right)=\sqrt{\frac{2}{3}}\left[\begin{array}{ccc} 1 & 0 & \frac{1}{\sqrt{2}}\\ -\frac{1}{2} & \frac{\sqrt{3}}{2} & \frac{1}{\sqrt{2}}\\ -\frac{1}{2} & -\frac{\sqrt{3}}{2} & \frac{1}{\sqrt{2}} \end{array}\right]\left[\begin{array}{c} i_{\alpha }\left(t\right)\\ i_{\beta }\left(t\right)\\ i_{\gamma }\left(t\right). \end{array}\right]$$

## 4. Implementation Process of Predictive Analytics Module

**Figure 4:**
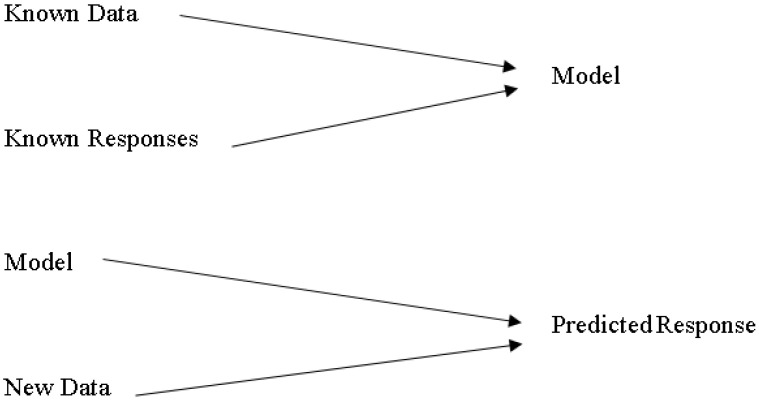
Predictive analytic based on supervised learning.

In the presence of uncertainty, supervised machine learning is used to create a model that predicts from evidence. When the adaptive algorithm identifies models in data, the computer “learns” through observation. When multiple observations are made, the computer improves its predictive performance. In particular, supervised learning ensures that the model generates a reasonable prediction of the response to the new data Figure 4.

After undertaking various steps of data processing, algorithm choosing, chosen method of validation, examination and updating of data until satisfied, the updated model is used for predictions. These steps are important before the preparation of data. All instructed instructional techniques start with input data, generally called *X*. The *X* rows depict an observation. The columns of *X* show a predictor or variable.

**Figure 5:**
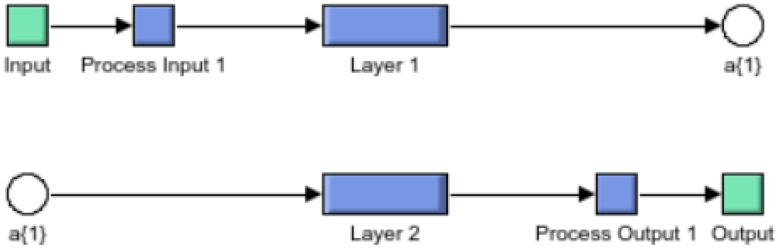
Predictive analytic-based shallow neural network.

The proposed ML-FFNN controller parameters in Table 2 are used for the learning process of the algorithm. The following hyper-parameters, such as activation function, batch size, epochs, and learning rate, are tuned to find the best parameters for the proposed ML-FFNN model. As a result, the proposed ML-FFNN model with the following hyper-meters (‘learning_rate’: 0.00025, ‘epochs’: 277, ‘batch_size’: 32, ‘activation’: ’sigmoid’) produced relatively better prediction results in terms of MAE, RMSE, and MSE score as compared to conventional (field oriented control scheme).

a. Shallow Neural Network: “Shallow” neural network is a term used to describe Neural Network (NN) that usually has only one hidden layer. Figure 5 shows a layered neural network architecture.

**Figure 6:**
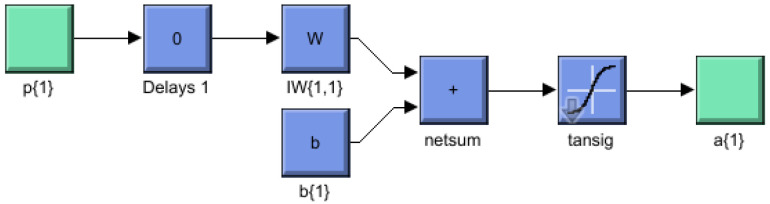
Input layer of shallow neural network.

The observations are directly associated with each of the neurons and the weight matrix as shown in Figure 6.

The layer comprises the weighing matrix, the bias vector and the summer, the activation function, and the output vector.

(5) a=f(Wp+b)

Vector *p* represent input data connected to each neuron over the weight matrix *w*, *b* indicates bias value and *f* is an activation function to determine output vector *a* for the summed weighted inputs. The elements from the input vector enter the network through the weight matrix as given in Equation (6):

(6) $$W=\left[\begin{array}{cccc} w_{1,1} & w_{1,2} & \cdots & w_{1,R}\\ w_{2,1} & w_{2,2} & \cdots & w_{2,R}\\ \vdots & \vdots & \; & \vdots \\ w_{S,1} & w_{S,2} & \cdots & w_{S,R}. \end{array}\right]$$

**Figure 7:**
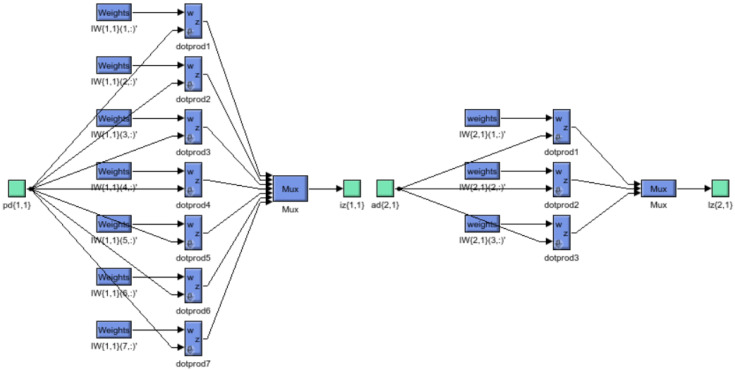
Schematic diagram of shallow NN with weights.

As mentioned before, the row elements of matrix *W* specify the targeted neuron connected with the weight. The input source for the weights is manifested in columns. Thus, the elements in “*w*”${}_{‘‘3,2"}$ indicate that the weight denotes the association with the third neuron from the latter source. The two-layer, 1−7−1 network. In this example, the first layer uses a log-sigmoid function and the second one uses a linear function as shown in Figure 7.

(7) $$f^{1}\left(n\right)=\frac{1}{1+e^{-n}}\;\;\mathrm{and}\;f^{2}\left(n\right)=n$$

(8) $$a^{1}=\log\mathrm{sig}\left(W^{1}p+b^{1}\right)$$

(9) $$a^{2}=\log\mathrm{sig}\left(W^{2}a^{1}+b^{2}\right).$$

When the network weights and biases are initialized, the network can be formed. The standard performance function of the feed-forward network is the average MSE among the output of a network and the destination output *t*.

b. The Back-propagation Algorithm In machine learning, output of one layer consider as the input of next layer as explained below:

(10) $$a^{m+1}=f^{m+1}\left(W^{m+1}a^{m}+b^{m+1}\right)\;\mathrm{for}\;m=0,1,\ldots ,M-1.$$

The total number of layers in ANN is *M*; the training inputs are connected to the neurons in a crisscross manner, which shows the beginning of Equation (10), because of the linear activation function, neurons in the last layer consider the output of ANN.

(11) $$a^{0}=p$$

(12) $$a=a^{M}.$$

For back-propagation batch descent optimization, a learning scheme is used for multi-layer ANN. This optimization algorithm shows examples, which are good for network behavior: $\left\{p_{1},t_{1}\right\},\left\{p_{2},t_{2}\right\},\ldots ,\left\{p_{Q},t_{Q}\right\}$ here $p_{Q}$ is an input to the ANN, and $t_{Q}$ is the related known responses at output, now after every epoch the ANN output compare with the known responses. The error produced after comparing known responses and the neural network output is minimized by adaptively changing the neural network weights.

(13) $$F\left(x\right)=E\left[e^{2}\right]=E\left[{\left(t-a\right)}^{2}\right].$$

In the case of machine learning, vector (*x*) consists of biases and weights.

(14) $$F\left(x\right)=E\left[e^{T}e\right]=E\left[{\left(t-a\right)}^{T}(t-a)\right].$$

The steepest descent algorithm for the approximate mean square error (stochastic gradient descent) is:

(15) $$w_{i,j}^{m}\left(k+1\right)=w_{i,j}^{m}\left(k\right)-\alpha \frac{\partial F}{\partial w_{i,j}^{m}}$$

(16) $$b_{i}^{m}\left(k+1\right)=b_{i}^{m}\left(k\right)-\alpha \frac{\partial F}{\partial b_{i}^{m}},$$

where α is the learning rate. The following equations are mainly used to take the differential of a shallow neural network. In the case of deep learning error, *e* is not the only direct function of weights in that layer; in this case, it is hard to compute their differentials.

(17) $$W\left(k+1\right)=W\left(k\right)+2\alpha e\left(k\right)p^{T}\left(k\right)$$

(18) b(k+1)=b(k)+2αe(k)(k).

Both *e* and *b* are vectors. We used the famous chain rule to calculate differentials of error, which is indirectly related to the weights of the hidden layer. Now let function *f* be only a function of polynomial *n*. Furthermore, to take differentials of function w.r.t 3rd variable which is weight *w* as shown in Equation (19).

(19) $$\frac{df(n(W))}{dw}=\frac{df(n)}{dn}\times \frac{dn(w)}{dw}.$$

If we consider this scenario as given in Equations (20) and (21):

(20) $$f\left(n\right)=e^{n},\;and\;n=2w,\;so\;that\;f\left(n\left(w\right)\right)=e^{2w}$$

(21) $$\frac{df(n(W))}{dw}=\frac{df(n)}{dn}\times \frac{dn(w)}{dw}=2e^{n}.$$

Next, we used this concept to find the derivatives in Equations (22) and (23):

(22) $$\frac{\partial F}{\partial w_{i,j}^{m}}=\frac{\partial F}{\partial n_{i}^{m}}\times \frac{\partial n_{i}^{m}}{\partial w_{i,j}^{m}}$$

(23) $$\frac{\partial F}{\partial b_{i}^{m}}=\frac{\partial F}{\partial n_{i}^{m}}\times \frac{\partial n_{i}^{m}}{\partial b_{i}^{m}}.$$

The input layer is a function of bias and with the weights of that layer, one can figure out the 2nd term in all of these equations straightforwardly.

(24) $$n_{i}^{m}=\sum_{j=1}^{S^{m-1}}W_{i,j}^{m}\;a_{j}^{m-1}+b_{i}^{m}.$$

Therefore, the sensitivity of *F* to changes in the *i*th element of the net input at layer *m*, then Equations (26) and (27) can be simplified to:

(25) $$\frac{\partial n_{i}^{m}}{\partial w_{i,j}^{m}}=a_{j}^{m-1}\;,\frac{\partial n_{i}^{m}}{\partial b_{i}^{m}}=1$$

(26) $$\frac{\partial F}{\partial w_{i,j}^{m}}=S_{m}^{i}$$

(27) $$\frac{\partial F}{\partial b_{i}^{m}}=S_{m}^{i}.\;$$

We can now express the approximate steepest descent algorithm as shown below in Equations (28) and(29).

(28) $$w_{i,j}^{m}\left(k+1\right)=w_{i,j}^{m}\left(k\right)-\alpha \;S_{i}^{m}\;a_{j}^{m-1}$$

(29) $$b_{i}^{m}\left(k+1\right)=b_{i}^{m}\left(k\right)-\alpha \;S_{i}^{m}.$$

This becomes in matrix form:

(30) $$W^{m}\left(k+1\right)=W^{m}\left(k\right)-\alpha S^{m}{\left(a^{m-1}\right)}^{T}$$

(31) $$b^{m}\left(k+1\right)=b^{m}\left(k\right)-\alpha S^{m},\;\mathrm{whereas}\;S^{m}=\frac{\partial F}{\partial n^{m}}.$$

## 5. Simulation Results and Analysis

The behavior of using several control techniques in this paper to enhance power quality, improve synchronization between renewable energy resources and the main electrical grid at PCC, and compensation of reactive power by using passive filters of the above electrical distribution system can be observed by studying the results obtained from various parameters after compensation.

### 5.1. Results of DCLMI

**Figure 8:**
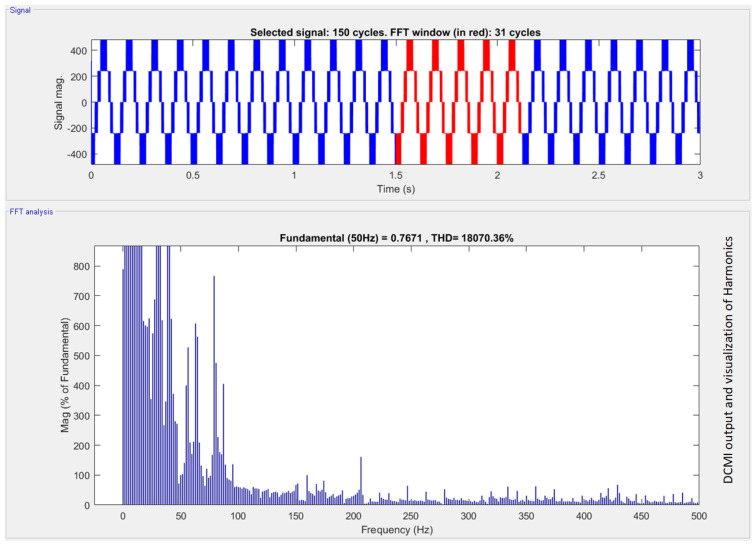
Visualization of diode clamped multilevel inverter and the harmonics produced due to the nonlinear devices used in it.

DCMI use dc voltage restorers’ diodes to limit the voltage stress of power IGBTs. A *k* level DCMI requires (2k−2) IGBT devices, (k−1) input dc voltage sources and (k−1)(k−2) clamping diodes in order to operate. $V_{dc}$ is the voltage across each diode and the switch. Three-phase DCMI is shown in Figure 2. The output waveform of DCMI is shown in Figure 8. The harmonics produced due to the non-linearities can be seen by applying the fast Fourier transform (FFT) analysis to the output signal of DCMI which are blue in color. The FFT analysis was performed only on the highlighted (red wave-forms) output cycles of the three-phase DCMI.

The magnitude of the harmonics when no passive filter is connected can be seen clearly. This is because the magnitude of the fundamental harmonic is the maximum, and the order of magnitude keeps decreasing with the increase in the harmonics.

**Figure 9:**
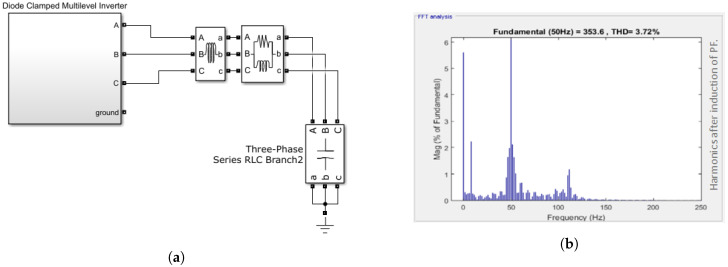
Harmonic compensation of micro-grid (DCMI) by connecting passive filters. (**a**) Schematic diagram of DCMI with passive filters. (**b**) Harmonic reduction after the connecting the passive filter.

After the passive filters are connected, the micro-grid, which is DCMI in this study, is used to compensate for harmonics. Figure 9 shows a clear decrease in the magnitude of the harmonics. There is a clear decrease in the harmonics, and the passive filters compensate for the harmonics as shown in Figure 9a. The comparison is made between Figure 9b and Figure 8. In that case, the amount of harmonics after the third harmonic is negligible in micro-grid containing passive filters. The micro-grid with passive filters also shows a magnitude decrease in fundamental harmonic and progressive harmonics.

### 5.2. Results at PCC

**Figure 10:**
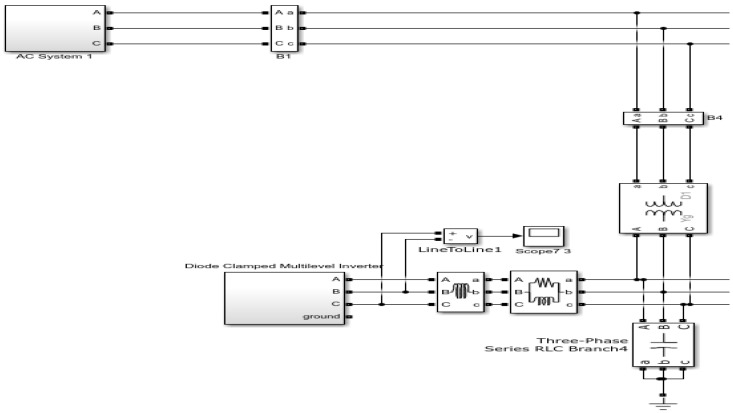
Connection of renewable energy sources with the micro-grid.

**Figure 11:**
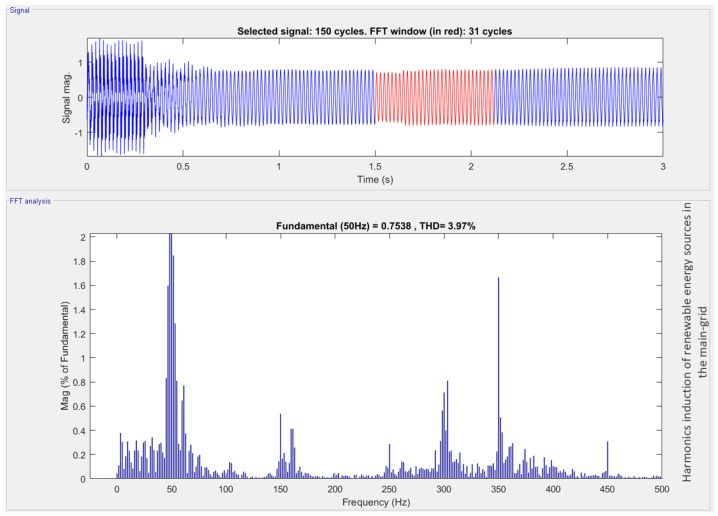
Result of harmonics after the induction of renewable energy sources in the main grid.

After connecting renewable energy resources with the main grid, as shown in Figure 10, harmonics are added to grid voltages due to nonlinear power devices used in the inverter. Figure 11 clearly shows the increase of harmonics in the main AC grid after adding renewable energy.

The harmonics produced due to the induction of renewable energy sources (RES) can be clearly seen by applying the FFT analysis on the micro-grid output signal after the RES connection. The FFT analysis was performed only on the highlighted output cycles.

From Figure 11, the addition of odd harmonics in the electrical system after the induction of RES is clearly seen.

**Figure 12:**
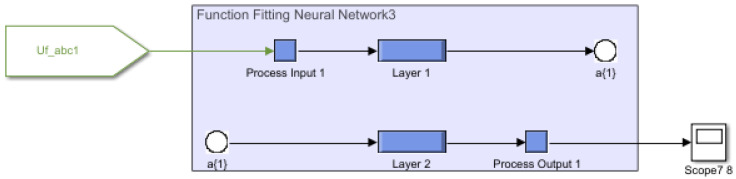
Harmonic compensation using ML-FFNN-based controller.

**Figure 13:**
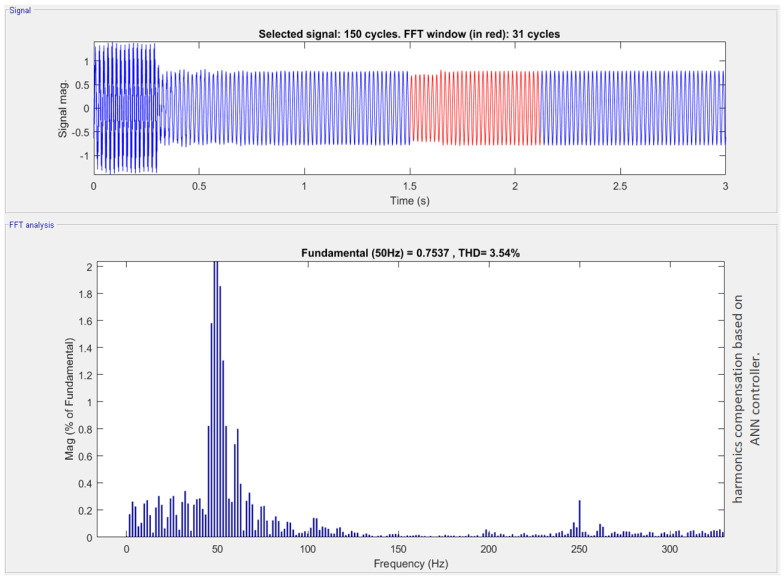
Result of harmonics compensation based on ML−FFNN controller.

By passing the above voltages through a MLFFNN controller, as shown in Figure 12 better results were achieved. In addition, harmonics in Figure 13 were significantly reduced by using the proposed AI assisted controller.

**Figure 14:**
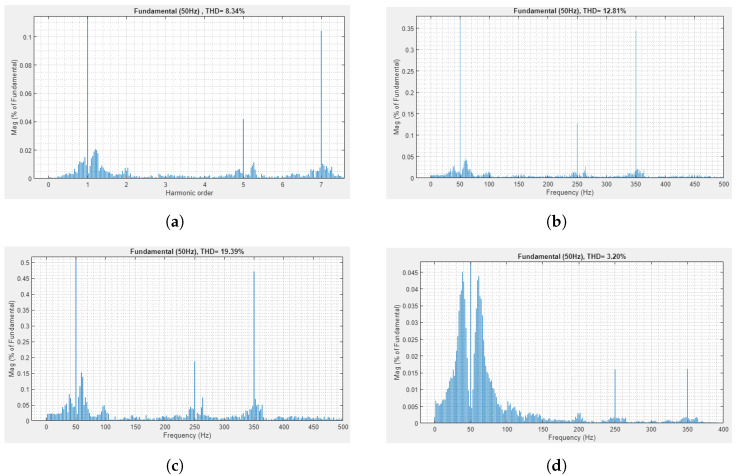
The comparison of harmonics at various stages of power electronic circuit. (**a**) Harmonics in three phase grid voltage. (**b**) Induction of harmonics after PCC. (**c**) Harmonics after PCC and load applying. (**d**) Harmonics in controller reference voltage.

Furthermore, Figure 14 highlights the harmonics at various stages of the power electronic circuit. Figure 14a denotes the harmonics in the three-phase grid voltages. Similarly, Figure 14b represents the harmonics after the point of common coupling. These harmonics are added to the power electronic circuit because of the non-linearities in the DCMLI. Furthermore, Figure 14c depicts the number of harmonics after applying the load at the grid voltages. On the other hand, Figure 14d shows the harmonics in reference voltages produced by the controller.

**Figure 15:**
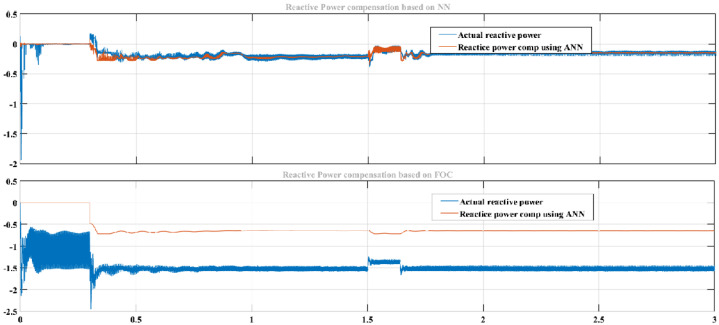
Reactive power compensation with ML−FFNN−based controller using step as a variation.

**Figure 16:**
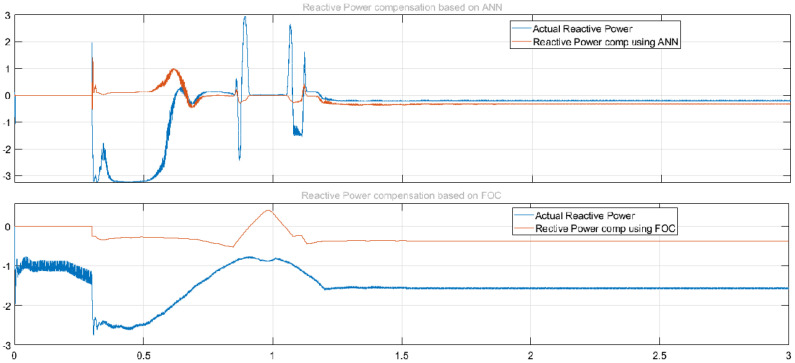
Comparison of ML−FFNN and FOC using modulation type of variation to check the performance of the proposed controller.

Similarly, better results were achieved in reducing reactive power with ML-FFNN than SRF, as shown in Figure 15. The upper half of the figure shows compensation of reactive power with ML-FFNN, and the lower half shows reactive power compensation with SRFC. Here the variation is used to check the performance of the proposed ML-FFNN assisted controller and compare the results with the baseline technique. Likewise, Figure 16 shows comparison of ML-FFNN and FOC using modulation as a type of variation to check the performance of the proposed controller. The variation timing in both experiments was chosen to be between 0.2 s and 1.2 s.

**Figure 17:**
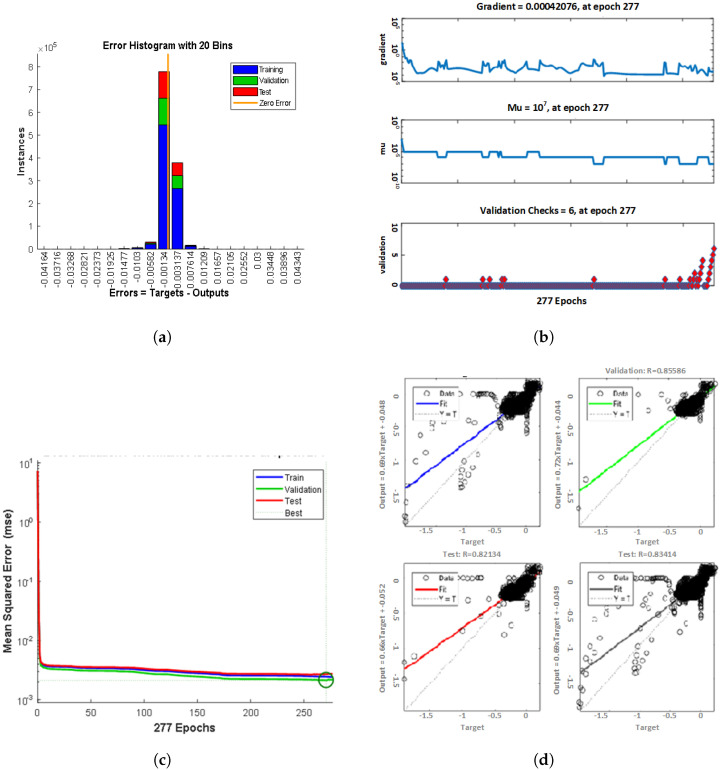
The evaluation of ML model using different evaluation metrics. ML–FFNN plots showing (**a**) Error histogram. (**b**) Regression Analysis. (**c**) Performance of training algorithm in reactive power compensation. (**d**) Mean square error.

The vertical columns shown in Figure 17 represent bins. One can calculate the total error range of shallow neural networks by noting the values of left and right most bins, which are −0.7534 and 0.3362, respectively. One can also find the bin’s width by finding the error range and dividing it by the total number of bins. Every column shows the number of examples from the neural network dataset, which lies in a specific bin. For instance, in the mid of the bar chart, the bin matching to the error of −0.0255 and the height of that bin for the validation dataset is $\left(0.9\times 10^{4}\right)$. It means that $\left(0.9\times 10^{4}\right)$ examples from the validation dataset have an error in the following range.

On the other hand, the regression plot below the bar-chart shows in Figure 17 how exactly your trained model fits the neural network dataset. The model prediction depends upon the value of R-squared; if the value is near 1, the model prediction is considered to be very good, and its badness shows how far the value of R-squared is from 1. On the other hand, if the value of R-squared is zero, the model fails to predict.

Now, this is only half of the judging performance. The other half is how validation is performed (is the model good at guessing out-of-sample values?) and how much regularization the network has performed.

The maximum number of epochs set were 277; if the validation error increased for more than six epochs, the training automatically stopped. Additionally, if one clicks on the performance plot in the neural network toolbox, it shows the graph of training errors, target data errors and validation data errors.

**Figure 18:**
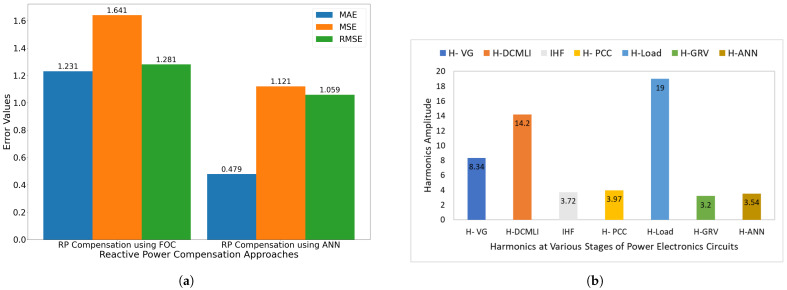
Comparison of ANN, FOC error and harmonics at various points of power electronic circuit. (**a**) Comparison of MLFFNN and FOC error for the suppression of reactive power. (**b**) Comparison of harmonics at various points of power electronic circuit.

The below results can be illustrated from the bar-graph as shown in Figure 18a. The performance of the conventional field-oriented control technique for the compensation of the reactive power is evaluated by using the evaluation metrics mean absolute error (MAE), mean square error (MSE), and root mean square error (RMSE). The result of MAE is 1.231, MSE 1.641, and RMSE 1.281 while analyzing the performance of FOC for the reactive power compensation. Similarly, the performance of the proposed technique ML-FFNN assisted controller for the compensation of the reactive power is evaluated using the same metrics. The proposed controller outperforms the baseline technique in all three evaluation metrics. The result of MAE was 0.479, MSE 1.121, and RMSE 1.059 while analyzing the performance of ML-FFNN for the compensation of reactive power. Figure 18 depicts the information regarding the harmonics at various stages of the power electronics circuit. The overall information regarding the performance for the suppression of harmonics using the proposed ML-FFNN assisted controller and its comparison with the FOC technique, along with the information on harmonics at other stages of an electrical system, can be seen in Figure 18.

**Figure 19:**
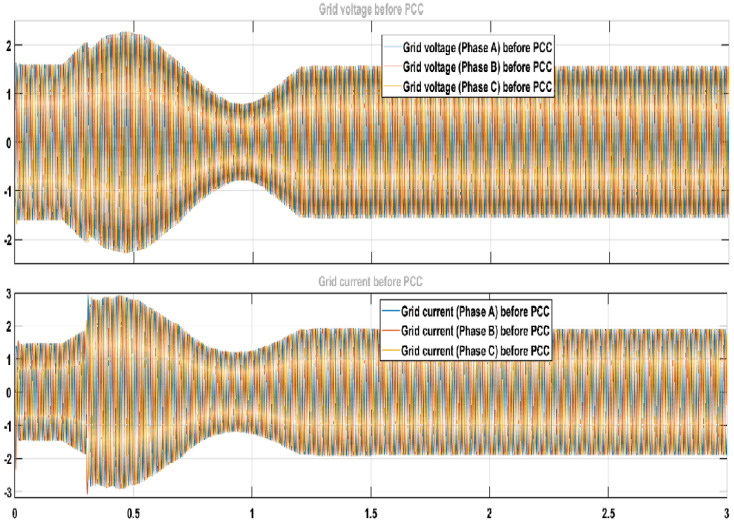
Three phase grid voltage and current before PCC.png.

**Figure 20:**
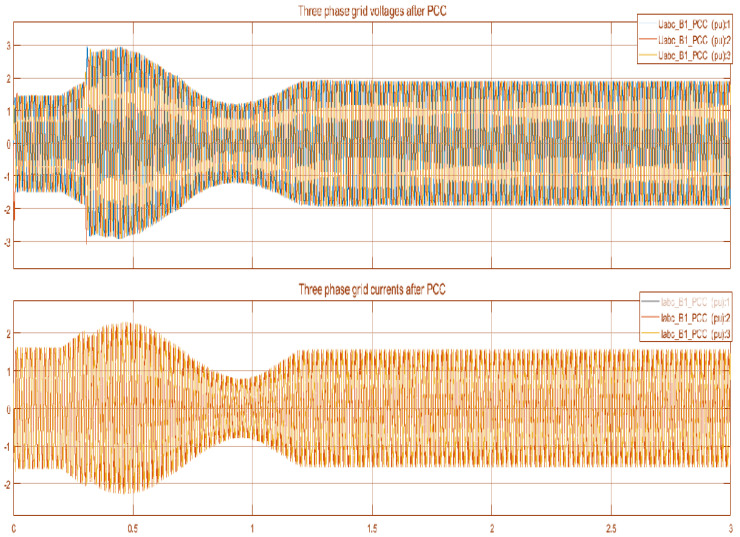
Three phase grid voltage and current after PCC.png.

Moreover, Figure 19 shows the three-phase voltage and current of the main electrical grid. Figure 20 represents the three-phase voltage and current of the main electrical grid after the PCC. The difference between the two figures shows the decrease in the three-phase grid current after PCC and improvement in three phase grid voltages because of the exchange of power into the main electrical grid.

### 5.3. Output of Dynamic Load Connecting to Main AC Grid

The three phases dynamic load block is a function of its +ve voltage sequence; both the exponents in the three-phase dynamic load block menu np and nq are fixed to 1 and also mentioned the $V_{min}$ is 0.7 pu. The below set of equations explains active and reactive power:

(32) $$If\;V>V_{min}$$

(33) $$P=P_{0}\,\left(\frac{V}{V_{0}}\right)\;;Q=Q_{0}\,\left(\frac{V}{V_{0}}\right)$$

On the other hand,

(34) $$If\;V<V_{min}$$

(35) $$P=P_{0}\,{\left(\frac{V}{V_{0}}\right)}^{2}\;;Q=Q_{0}\,{\left(\frac{V}{V_{0}}\right)}^{2}.$$

**Figure 21:**
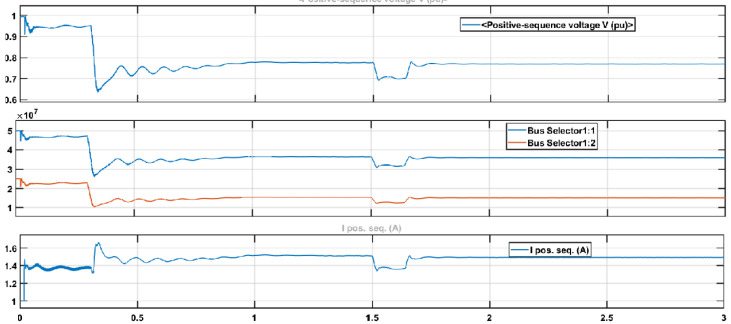
Three phase dynamic and nonlinear load connected to main AC grid.

The constant load current is shown in Figure 21 if a voltage is greater than 0.7 pu. If the voltage decreases below the mentioned voltage value, then it behaves as a constant impedance load.

One can vary active *P* and reactive *Q* power as a voltage function. The three-phase voltage source block is used to control the source internal voltage. By clicking on the menu, one can find out that the definite type of amplitude variation is a step. Additionally, the +ve sequence voltage changes between the time of one’s choice; in this case, it changes between (1.5 to 1.7) pu. In the start, the voltage of the source is 1 pu.

**Figure 22:**
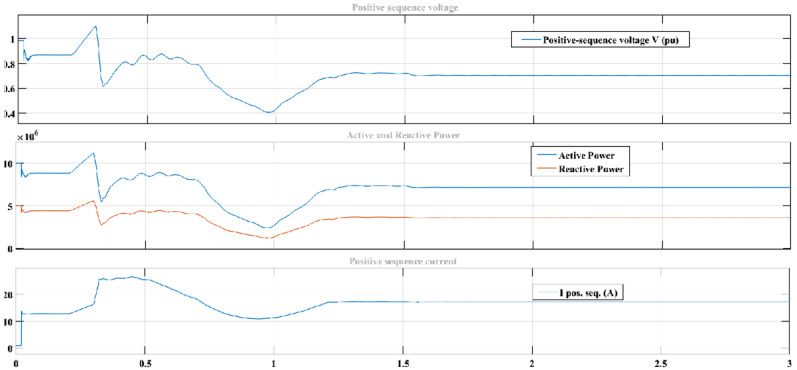
Three phase dynamic and nonlinear load connected to main AC grid to observe the positive sequence voltage and current using modulation as variation type.

Similarly, Figure 22 shows the variation in positive sequence voltage and current. The variation timing considered for this experiment is from 0.2 to 1.3. The type of variation considered is sinusoid modulation.

In both the figures discussed above, the top plot shows the positive sequence voltage, the middle figure shows active and reactive power, and the bottom plot shows positive sequence current.

## 6. Limitations

The major limitation of this study is that the proposed ML-FFNN model requires reliable data to learn the hidden patterns to produce a generalized inference model for the compensation of reactive power controller. Without the availability of the data, one cannot use the proposed learning-based controller for the reactive power compensation problem. In addition, our proposed ML-FFNN assisted controller used a trial and error method to determine hyper-parameters, such as learning rate, activation function, optimizer, etc. This will affect the model performance as well as increase the computational overhead of the proposed ML-FFNN assisted controller. Furthermore, our proposed approach used gradient descent to maintain a learning rate per parameter, which may cause to trap the ML-FFNN model in the local minimum problem. Moreover, all these limitations will be tackled in future studies, as discussed in the next section, to enhance the performance and efficiency of the proposed research study.

## 7. Conclusions and Future Work

This paper presented a detailed comparison of the SRF and AI-based control techniques for the proposed multi-level inverter. ML-FFNN was employed to predict non-linearities to minimize the error between target and reference voltages. The stochastic gradient descent optimization algorithm involves an optimal weight and biases tuning process to attain optimized network performance and produce a generalized model. The back-propagation method is adopted for gradient computation to improve the overall performance of the proposed model. Furthermore, MSE, MAE, and RMSE were used as evaluation metrics to assess the performance of the proposed prediction model by estimating the average squared error between actual and estimated output. The simulation-based results confirmed that the proposed ML-FFNN-based harmonic and reactive power control technique performs 0.752 better in terms of MAE, 0.52 for the case of MSE, and 0.222 when evaluating based on the RMSE. In addition, passive filters were used on both the input and load sides to minimize voltage and current ripples. FFT analysis compares multi-level inverters, indicating the considerable reduction of harmonics and total harmonic distortion in nine-level inverters. Furthermore, increasing inverter levels improves output waveform quality, reducing filter size, stress on inverter switches, and the cost of the multi-level inverter. The simulation results indicate that the proposed ML-FFNN produces satisfactory results for the compensation of reactive power as compared to the synchronous reference frame control technique. Moreover, experimental results indicate that our proposed ML-FFNN-based control scheme is stable over a wide range of operations with minimum transients.

Moreover, in future work, our proposed ML-FFNN-based compensation of reactive power can be enhanced by considering historical data from different micro-grids to produce an efficient generalized model. Furthermore, hyper-parameters’ optimization techniques, such as random search, hyperband, etc., will be considered to improve the performance. In addition, evolutionary techniques will be employed to optimize and tune the weights of our proposed ML-FFNN model to improve the accuracy and generalize the model to converge quickly as compared to the baseline learning model.

## Figures and Tables

**Table 1:** Grid, Inverter, and PI controller parameters.

Parameters	Value
Nominal AC Grid Voltage	230 kV
Fundamental Grid Frequency	50 Hz
Grid Base Power	200 MW
Switching Frequency	3.33 kHz
Transformer Rated Power	200 MVA
Winding 1 Parameters	480 × 0.915
Winding 2 Parameters	230 kV
DCMLI output Voltage	480 V
Inverter Base Power	10 kW
Shunt Capacitence	241 µF
Series Inductance	150.02 mH
Current Regulator	Kp = 0.57, Ki = 6.08
Reactive and Active Power Control	Kp = 3.02, Ki = 2.92
DC voltage Balance Control	Kp = 0.019, Ki = 0.027
Active Load Applied at Grid Side	1 MW
Nonlinear Load	Diode Bridge Rectifier

**Table 2:** ANN Controller Parameters.

ANN Controller Parameters	Value
Activation Function	Sigmoid
Number of layers	2
Learning rate	0.00025
Optimizer	SGD
Loss function	MSE
Metrics	R2 score
Training Epoch	277
Layer composition	Weight matrix, bias vector, summer, activation function, output vector

## Data Availability

Not applicable.
